# L-shaped association between fasting blood glucose and urea in a non-diabetic population

**DOI:** 10.3389/fnut.2025.1504855

**Published:** 2025-03-24

**Authors:** Chenguang Wu, Zhenyan Xu, Xin Chen, Hualong Liu, Yuliang Chen, Jiaxing Huang, Teng Lu, Zixi Huang

**Affiliations:** ^1^Department of Cardiovascular Medicine, The Second Affiliated Hospital, Jiangxi Medical College, Nanchang University, Nanchang, China; ^2^Department of Health Care, The Second Affiliated Hospital, Jiangxi Medical College, Nanchang University, Nanchang, China; ^3^Department of General Practice, The Second Affiliated Hospital, Jiangxi Medical College, Nanchang University, Nanchang, China

**Keywords:** urea, fasting blood glucose, non-diabetic population, protein, association

## Abstract

**Background:**

The relationship between fasting blood glucose and urea in non-diabetic individuals is still unclear. This study aimed to evaluate the association between fasting blood glucose and urea in a non-diabetic population.

**Methods:**

Data from a cohort of non-diabetic individuals were collected from the 2009 China Health and Nutrition Survey dataset. We performed smooth curve and two piecewise linear regression analyses to assess the association between fasting blood glucose and urea in this non-diabetic population.

**Results:**

Data from a total of 7,596 adult participants without diabetes were included in this study; the mean age of the participants was 50.2 years, and 46.4% were male. There was an L-shaped relationship between fasting blood glucose and urea, and the inflection point of fasting blood glucose was 4.6 mmol/L. After adjusting for potential confounders, we found a negative correlation between fasting blood glucose and urea up to the inflection point (β = −0.3, 95% CI −0.5 to −0.2, *P* < 0.001), but beyond the inflection point, this relationship disappeared (β = 0.0, 95% CI −0.1 to 0.1 *P* = 0.848). In the group with lower fasting blood glucose (fasting blood glucose <4.6 mmol/L), smoking (interaction *P* = 0.037) and alcohol consumption (interaction *P* = 0.001) influenced the relationship between fasting blood glucose and urea.

**Conclusions:**

The results suggest that lower fasting blood glucose was associated with higher urea in non-diabetic individuals with fasting blood glucose <4.6 mmol/L, revealing an L-shaped association between fasting blood glucose and urea.

## 1 Introduction

Urea is the terminal product of protein catabolism; as a water-soluble substance, it can be excreted through the kidneys (1). The process of urea production and excretion is altered under several conditions, such as changes in protein metabolism and kidney disease (2). Increased protein consumption leads to an increased concentration of urea in the blood (3). In addition, as the kidneys play an essential role in the excretion of urea, prerenal, intrarenal and postrenal diseases can lead to a significant increase in urea (2). Therefore, urea levels are widely used in the clinical evaluation of renal function and can be a useful biomarker for predicting the progression of kidney diseases (4). Recent studies have demonstrated that the probabilities of diabetes mellitus and diabetic retinopathy increase by 7% and 12%, respectively, for every 1 mmol/L increase in urea (5, 6). Many studies have shown a significant association between high urea levels and an increased risk of mortality in patients with heart failure, chronic kidney disease, acute ischemic stroke, or acute pulmonary embolism (4, 7–9). Increased urea negatively affects the cardiovascular, endocrine, and urinary systems, suggesting the importance of urea (10, 11).

Carbohydrate and protein metabolism are closely associated and provide the necessary energy for the human body. In addition, glucose always participates in the process of renal injury, and high or low blood glucose levels can damage renal function through multiple pathways (12–14). At present, studies on the association between blood glucose and urea have focused mostly on patients with diabetes. It has been shown that urea levels are significantly positively associated with both short-term and long-term glycemic variability in patients with diabetes mellitus (15). Moreover, urea levels were significantly higher in patients with poor glycemic control during 12 years of follow-up (16). In non-diabetic individuals, one study reported that a decrease in blood glucose caused by fasting led to an increase in the urea concentration (17), whereas another study reported that a decrease in blood glucose led to a decrease in the urea concentration (18). Therefore, the relationship between blood glucose and urea in non-diabetic individuals is still unclear. Therefore, the aim of this study was to further evaluate the association between blood glucose and urea in a non-diabetic population to provide additional evidence.

## 2 Methods

### 2.1 Data source and study population

The data that were analyzed were extracted from the 2009 China Health and Nutrition Survey (CHNS) dataset. The CHNS is a large-scale longitudinal survey designed to provide economic, sociological, demographic, dietary, health and physical activity data to measure key public health risk factors. Fasting blood samples were collected for the first time in 2009. Informed consent was obtained from the participants involved in the database. Details of the survey were published previously (19), and all the data are available to researchers for free on the following website: http://www.cpc.unc.edu/projects/china.

### 2.2 Study population

The 2009 CHNS dataset of 12,009 individuals was initially included in this study. The estimated glomerular filtration rate (eGFR) was calculated via the Modification of Diet in Renal Disease equation [*eGFR* = 175 × *Scr*^−1.154^ × *age*^−0.203^ × 1.212 (*if black*) × 0.742(*if female*)], where the eGFR is expressed in mL/min/1.73 m^2^ and the serum creatinine (Scr) concentration is expressed in mg/dL (20). The body mass index (BMI) was calculated as body weight (kilograms) divided by the square of height (meters). Individual dietary data were assessed by considering three consecutive days of 24-h dietary recall by trained nutritionists. The participants were asked to avoid fierce exercise or high mental pressure before the collection of plasma samples to measure fasting glucose levels (participant avoided eating for at least 8 h but not more than 16 h). Additional details were described elsewhere (19). The exclusion criteria were as follows: (1) aged < 18 years; (2) pregnant; (3) missing information about laboratory variables (urea or fasting blood glucose) and medical history (diabetes mellitus); (4) diagnosed with diabetes on the basis of the guidelines or had a history of diabetes (21); and (5) missing information about the covariates mentioned below.

A total of 7,596 non-diabetic participants were included in the analysis. To handle missing data, we exclude variables with more than 25% missing values. For normally distributed continuous variables, the missing values were replaced by the mean value, and for continuous variables with skewed distributions, the missing values were replaced by the median value (22). There were no missing dichotomous variables in this study.

### 2.3 Covariates

Variables that may be related to fasting blood glucose and urea were adjusted to improve the reliability of the results. This study covered six dimensions of variables, including demographic characteristics, such as age, sex, and BMI; vital indices, such as systolic blood pressure and diastolic blood pressure; medical history of hypertension, stroke, and myocardial infarction; lifestyle factors, such as smoking, alcohol consumption, tea consumption, coffee consumption, soft/sugared fruit drink consumption; dietary factors, such as carbohydrate intake, fat intake, dietary protein intake, and total energy intake; and laboratory variables, such as uric acid, creatinine, alanine transaminase, high-density lipoprotein cholesterol, low-density lipoprotein cholesterol, triglyceride, total cholesterol, total protein, and albumin.

### 2.4 Statistical analysis

The data are presented as the mean [standard deviation (SD)] for normally distributed data, as the median [interquartile range (IQR)] for skewed data, and categorical variables are presented as numbers (percentages). For continuous variables, we used independent *t* tests (normal distribution) or Kruskal–Wallis rank–sum tests (skewed distribution) to compare the differences between groups. For categorical variables, chi-square tests were used to compare differences in proportions between groups.

First, we constructed a smooth curve to estimate the relationship between fasting blood glucose and urea after adjustment for the covariates listed above. We also constructed smooth curves for the relationships of fasting glucose with creatinine and the eGFR. Second, the threshold value was obtained on the basis of the fitting results of the smooth curve and the log likelihood ratio test. Then, according to the inflection point, a two-piecewise linear regression model was performed to evaluate the non-linear relationship between fasting blood glucose and urea. We constructed three models to increase the reliability of the results (Crude Model: no covariates were adjusted; Model 1: adjusted for only age and sex; and Model 2: adjusted for all covariates). Finally, stratified analysis and interaction analysis were performed to evaluate the potential modifiers of the association between fasting blood glucose and urea. Each stratification was adjusted for all covariates except the stratification factor itself.

All the analyses were performed with the statistical software Stata 17.0 (College Station, TX) and EmpowerStats (X&Y Solutions, Inc. Boston, MA). *P* < 0.05 was considered statistically significant in all analyses.

## 3 Results

### 3.1 Baseline characteristics

**Figure 1:**
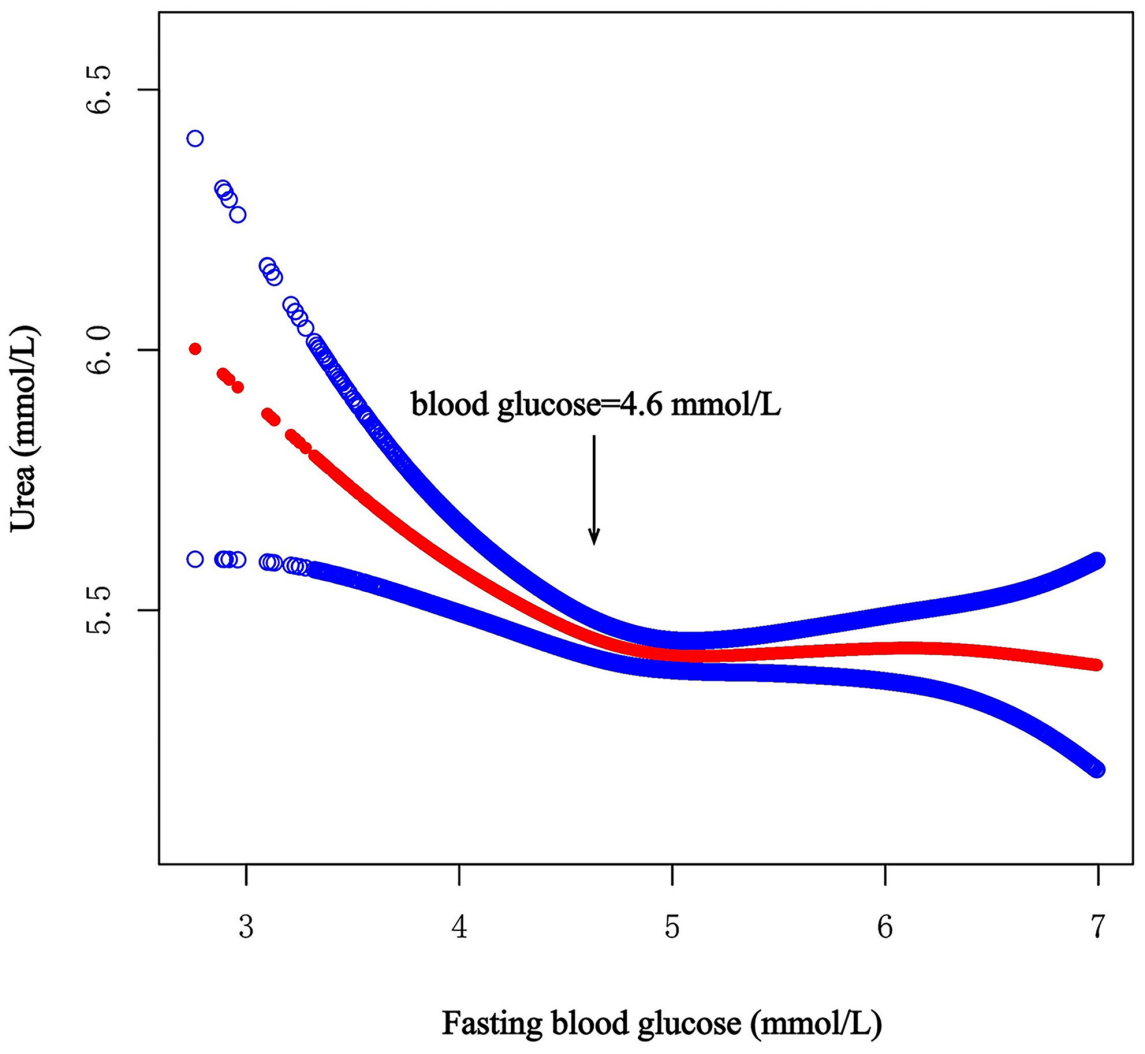
Smooth curve on association between fasting blood glucose and urea. The red curve represents the smooth curve fit between variables. The Blue curves represent the 95% confidence interval around the fitted trend.

A total of 7,596 participants, including 3,521 men and 4,075 women, were enrolled in this study. We calculated the threshold value, as shown in Figure 1. All the participants were divided into two groups according to the inflection point of glucose (fasting blood glucose <4.6 mmol/L or fasting blood glucose ≥4.6 mmol/L). Table 1 lists the baseline characteristics of all the enrolled participants, including demographic characteristics, vital signs, medical history, lifestyle factors, dietary factors and laboratory variables. Table 1 also shows the differences between the two groups.

**Table 1:** Clinical characteristics of study participants.

**Variables**	**Total**	**Glucose < 4.6**	**Glucose ≥ 4.6**	***P*-value**
N	7,596	1,560	6,036	
Age, mean (SD), years	50.2 (15.0)	46.1 (15.7)	51.2 (14.7)	<0.001
eGFR, mean (SD), mL/min/1.73 m^2^	72.2 (14.9)	74.9 (14.7)	71.4 (14.9)	<0.001
SBP, mean (SD), mmHg	124.0 (18.7)	120.4 (17.7)	124.9 (18.8)	<0.001
DBP, mean (SD), mmHg	80.2 (11.3)	78.9 (11.4)	80.6 (11.2)	<0.001
Uric acid, mean (SD), umol/L	304.3 (97.6)	289.5 (104.8)	308.1 (95.3)	<0.001
Creatinine, mean (SD), umol/L	87.2 (23.5)	85.8 (17.4)	87.6 (24.9)	0.008
HDL, mean (SD), mmol/L	1.4 (0.5)	1.4 (0.4)	1.4 (0.5)	0.476
LDL, mean (SD), mmol/L	3.0 (1.0)	2.7 (0.8)	3.0 (1.0)	<0.001
Triglyceride, median (Q1–Q3), mmol/L	1.2 (0.8–1.9)	1.0 (0.7–1.5)	1.3 (0.9–2.0)	<0.001
Total cholesterol, mean (SD), mmol/L	4.8 (1.0)	4.5 (0.9)	4.9 (1.0)	<0.001
Total protein, mean (SD), g/L	77.2 (5.1)	75.8 (4.9)	77.6 (5.1)	<0.001
Albumin, mean (SD), g/L	47.5 (3.4)	46.7 (3.3)	47.7 (3.4)	<0.001
calorie, mean (SD), kcal/d	2,139.1 (654.7)	2,134.8 (626.7)	2,140.2 (661.8)	0.773
Carbohydrate, mean (SD), g/d	296.3 (101.8)	303.4 (103.3)	294.4 (101.3)	0.002
Fat, median (Q1–Q3), g/d	68.9 (49.2–93.7)	66.8 (48.2–90.7)	69.6 (49.5–94.5)	0.006
Protein, mean (SD), g/d	65.9 (22.9)	65.4 (22.4)	66.1 (23.0)	0.284
BMI, mean (SD), kg/m^2^	23.2 (3.4)	22.4 (3.1)	23.5 (3.4)	<0.001
Apolipoprotein A, mean (SD), g/L	1.2 (0.4)	1.1 (0.3)	1.2 (0.4)	<0.001
Lipoprotein a, median (Q1–Q3), mg/L	79.5 (41.0–170.0)	85.0 (43.0–178.0)	78.0 (40.8–168.0)	0.041
C-reactive protein, median (Q1–Q3), mg/L	1.0 (0.0–2.0)	1.0 (0.0–2.0)	1.0 (1.0–2.0)	<0.001
Apolipoprotein B, mean (SD), g/L	0.9 (0.3)	0.8 (0.2)	0.9 (0.3)	<0.001
Serum magnesium, mean (SD), mmol/L	0.9 (0.1)	0.9 (0.1)	0.9 (0.1)	<0.001
Ferritin, median (Q1–Q3), ng/mL	77.2 (38.5–141.0)	63.6 (31.1–120.4)	80.8 (41.2–146.5)	<0.001
Insulin, median (Q1–Q3), IU/mL	10.1 (7.2–14.4)	8.5 (6.2–11.6)	10.6 (7.5–15.1)	<0.001
Hemoglobin, mean (SD), g/L	141.1 (20.5)	140.5 (20.2)	141.2 (20.5)	0.191
White blood cells (×10∧9 cells/L), mean (SD)	6.2 (1.9)	6.4 (1.8)	6.2 (1.9)	0.003
Red blood cells (×10∧12 cells/L), mean (SD)	4.7 (0.7)	4.7 (0.7)	4.7 (0.7)	0.553
Platelet count (×10∧9 cells/L), mean (SD)	213.2 (68.5)	219.2 (69.2)	211.7 (68.3)	<0.001
HbA1c, mean (SD), %	5.5 (0.6)	5.3 (0.5)	5.5 (0.6)	<0.001
ALT, median (Q1–Q3), U/L	18.0 (13.0–26.0)	16.0 (12.0–23.0)	19.0 (13.0–27.0)	<0.001
Transferrin, mean (SD), mg/dL	286.9 (54.4)	284.0 (56.5)	287.6 (53.8)	0.022
STR median (Q1–Q3), mg/L	1.3 (1.1–1.6)	1.3 (1.1–1.7)	1.3 (1.1–1.6)	0.707
**Gender**				0.535
Male	3,521 (46.4%)	734 (47.1%)	2,787 (46.2%)	
Female	4,075 (53.6%)	826 (52.9%)	3,249 (53.8%)	
**Smoke**				<0.001
No	5,231 (68.9%)	1,013 (64.9%)	4,218 (69.9%)	
Yes	2,365 (31.1%)	547 (35.1%)	1,818 (30.1%)	
**Tea consumption**				<0.001
No	4,954 (65.2%)	1,134 (72.7%)	3,820 (63.3%)	
Yes	2,642 (34.8%)	426 (27.3%)	2,216 (36.7%)	
**Coffee consumption**				0.362
No	7,397 (97.4%)	1,514 (97.1%)	5,883 (97.5%)	
Yes	199 (2.6%)	46 (2.9%)	153 (2.5%)	
**Alcohol consumption**				0.577
No	5,104 (67.2%)	1,039 (66.6%)	4,065 (67.3%)	
Yes	2,492 (32.8%)	521 (33.4%)	1,971 (32.7%)	
**DSFD consumption**				<0.001
No	4,883 (64.3%)	929 (59.6%)	3,954 (65.5%)	
Yes	2,713 (35.7%)	631 (40.4%)	2,082 (34.5%)	
**Hypertension**				<0.001
No	6,714 (88.4%)	1,447 (92.8%)	5,267 (87.3%)	
Yes	882 (11.6%)	113 (7.2%)	769 (12.7%)	
**Ischaemic heart disease**				0.038
No	7,535 (99.2%)	1,554 (99.6%)	5,981 (99.1%)	
Yes	61 (0.8%)	6 (0.4%)	55 (0.9%)	
**Stroke**				0.115
No	7,503 (98.8%)	1,547 (99.2%)	5,956 (98.7%)	
Yes	93 (1.2%)	13 (0.8%)	80 (1.3%)	

SBP, systolic blood pressure; DBP, diastolic blood pressure; HDL, high-density lipoprotein cholesterol; LDL, low-density lipoprotein cholesterol; BMI, body mass index; HbA1c, glycosylated hemoglobin; ALT, alanine transaminase; STR, soluble transferrin receptor; DSFD, drinking soft/sugared fruit drink. Data are presented as mean ± SD, median (IQR), or %.

### 3.2 L-shaped association between blood glucose and urea

To analyze the non-linear association between fasting blood glucose and urea, three models were constructed, and the results are shown in Table 2.

**Table 2:** Two-piecewise linear regression for relationship between fasting blood glucose and urea.

**Variables**		**β**	**β 95%CI**	***P*-value**
Crude Model	Inflection point (4.6)			
	<4.6	−0.4	−0.6, −0.2	<0.001
	≥4.6	0.3	0.2, 0.3	<0.001
	Likelihood ratio test			<0.001
Model 1	Inflection point (4.6)			
	<4.6	−0.4	−0.6, −0.2	<0.001
	≥4.6	0.1	0.0, 0.2	0.001
	Likelihood ratio test			<0.001
Model 2	Inflection point (4.6)			
	<4.6	−0.3	−0.5, −0.2	<0.001
	≥4.6	0.0	−0.1, 0.1	0.848
	Likelihood ratio test			0.001

After full adjustment for covariates by Model 2, the smooth curve revealed an L-shaped relationship between fasting blood glucose and urea (*P* = 0.011) (Figure 1). In the threshold effect analysis using two piecewise regression models, we found evidence of non-linear associations between fasting blood glucose and urea (P for the log-likelihood ratio test = 0.001). After adjusting for the above covariates, the cut-off value for fasting blood glucose was 4.6 (Table 2). In participants with fasting blood glucose < 4.6 mmol/L, urea decreased with increasing fasting blood glucose (β = −0.3, 95% CI: −0.5 to −0.2, *P* < 0.001). However, when fasting blood glucose was ≥4.6 mmol/L, fasting blood glucose was not associated with urea (β = 0.0, 95% CI: −0.1 to 0.1, *P* = 0.848). Other model details are shown in Table 2. In addition, we also constructed smooth curves to estimate the relationships of fasting blood glucose with creatinine and the eGFR. The results revealed that creatinine and the eGFR were not significantly associated with blood glucose (*P* > 0.05) (Supplementary Figures S1, S2).

### 3.3 Stratified analysis and interaction analysis

To further explore the relationship between glucose and urea, we performed a stratified analysis using different stratification variables, which were included in Model 2, except the stratification factor itself. The results are shown in Table 3. We found interactions for smoking (interaction *P* = 0.037) and alcohol consumption (interaction *P* = 0.001) in the group with low fasting blood glucose (fasting blood glucose < 4.6 mmol/L). In this group, the negative correlation between fasting blood glucose and urea disappeared in participants who did not smoke (*P* = 0.795) or consume alcohol (*P* = 0.747).

**Table 3:** Stratified analysis and interaction analysis.

**Subgroup**	**Glucose**<**4.6**			**Glucose** ≥ **4.6**		
	β**, 95%CI**	**P**	**Interaction P**	β**, 95%CI**	**P**	**Interaction P**
**Gender**						
Male	−0.3 (−0.7, 0.0)	0.065	0.523	−0.0 (−0.1, 0.1)	0.491	0.055
Female	−0.2 (−0.5, 0.2)	0.376		0.1 (−0.0, 0.2)	0.052	
**Smoke**						
No	−0.0 (−0.3, 0.3)	0.795	**0.037**	0.1 (−0.0, 0.2)	0.130	0.157
Yes	−0.6 (−0.7, −0.2)	0.005		−0.0 (−0.2, 0.1)	0.552	
**Tea consumption**						
No	−0.2 (−0.5, 0.1)	0.112	0.838	0.1 (−0.2, 0.2)	0.138	0.197
Yes	−0.3 (−0.8, 0.2)	0.252		−0.0 (−0.1, 0.1)	0.696	
**Coffee consumption**						
No	−0.3 (−0.5, −0.0)	0.040	0.327	0.0 (−0.0, 0.1)	0.384	0.991
Yes	0.7 (−1.2, 2.26)	0.476		0.0 (−0.4, 0.5)	0.884	
**Alcohol consumption**						
No	0.0 (−0.3, 0.4)	0.747	**0.001**	0.0 (−0.1, 0.1)	0.634	0.645
Yes	−0.8 (−1.2, −0.4)	<0.001		0.1 (−0.1, 0.2)	0.369	
**DSFD consumption**						
No	−0.1 (−0.4, 0.2)	0.565	0.086	0.0 (−0.1, 0.1)	0.628	0.617
Yes	−0.5 (−0.9, −0.1)	0.012		0.1 (−0.1, 0.2)	0.358	
**Hypertension**						
No	−0.2 (−0.5, 0.0)	0.082	0.572	0.0 (−0.1, 0.1)	0.647	0.333
Yes	−0.5 (−1.4, 0.4)	0.295		0.1 (−0.1, 0.3)	0.215	
**Ischaemic heart disease**						
No	−0.2 (−0.5, 0.0)	0.056	0.559	0.0 (−0.0, 0.1)	0.384	0.956
Yes	−1.6 (−6.2, 3.0)	0.496		0.1 (−0.6, 0.7)	0.878	
**Stroke**						
No	−0.2 (−0.5, 0.0)	0.068	0.292	0.0 (−0.0, 0.1)	0.453	0.287
Yes	−1.4 (−3.6, 0.8)	0.210		0.3 (−0.2, 0.9)	0.246	

DSFD, drinking soft/sugared fruit drinks. Bold values indicate statistical significance (*P* < 0.05).

## 4 Discussion

We first found an L-shaped association between fasting blood glucose and urea in the non-diabetic population after adjusting for important identified confounders, and the cut-off for the curve was a fasting blood glucose level of 4.6 mmol/L, which is different from the previous definition of hypoglycemia (21). In addition, smoking and alcohol consumption were found to affect the association between fasting blood glucose and urea in individuals with fasting blood glucose levels lower than 4.6 mmol/L. Instead, the negative correlation between fasting blood glucose and urea disappeared in participants who did not smoke or consume alcohol.

Gluconeogenesis can be an important source of glucose when blood glucose levels decrease. This process plays a critical role in glucose homeostasis, allowing the body to synthesize glucose from non-carbohydrate substances, such as glucogenic amino acids (23). Amino acid catabolism produces a large amount of nitrogen, and gluconeogenic enzymes are expressed together with urea cycle enzymes in the liver, which means that amino acid gluconeogenesis is closely associated with ureagenesis (24). Alanine is converted to pyruvate and glutamate by alanine aminotransferase in the liver, and glutamate eliminates toxic ammonium and synthesizes excess urea through the urea cycle (23, 25). This may be one of the reasons why the urea concentration increases when blood glucose levels are low.

In addition, urea synthesis is regulated by hormones, such as glucagon (2). When the blood glucose concentration decreases, the secretion of glucagon, which plays an important role in nitrogen metabolism, can be stimulated (26). Glucagon affects urea synthesis in several ways. Glucagon stimulates gluconeogenesis to produce glucose, and during this process, the nitrogen of amino acids is excreted mainly in the form of urea (26). Additionally, glucagon increases the activity of ornithine cycle enzymes (27). Furthermore, glucagon stimulates an increase in the N-acetylglutamate concentration, which is an essential allosteric activator of the urea cycle (28, 29). Moreover, increased glucagon leads to the activation of AMP-activated protein kinase, which is involved in inhibiting protein synthesis and stimulating proteolysis and ureagenesis (30, 31). In various animal models, increased plasma glucagon concentrations increase amino acid catabolism and urea synthesis, whereas glucagon deficiency decreases amino acid clearance and urea production (30, 32). In a study of healthy male adults, hypoglycemia was associated with a doubling of glucagon levels and a significant increase in hepatic urea production (33). Another study reported increased hepatic urea synthesis during glucagon infusion in healthy adults (34).

Clearly, renal excretion of urea is an important determinant of urea concentration, and an elevated urea concentration is a marker of renal dysfunction and injury (4). Glucose levels are closely associated with renal injury, including hyperglycemia and hypoglycemia (14, 35). To investigate whether the L-shaped relationship between fasting blood glucose and urea in the non-diabetic population occurred due to renal dysfunction caused by glucose, we also implemented smooth curves to estimate the relationships of fasting blood glucose with creatinine and the eGFR. The results revealed that creatinine and the eGFR were not significantly associated with blood glucose. This finding indicates that in non-diabetic individuals, blood glucose primarily affects urea synthesis through protein metabolism rather than affecting urea excretion through renal injury. Further mechanistic studies, such as animal models or *in vitro* experiments are essential to elucidate underlying biological pathways.

Notably, the urea concentration may be a confounding effect of age and nutritional status, especially protein intake (4, 36). Therefore, we adjusted for multiple covariates, including protein intake, total serum protein, renal function, age and other laboratory variables, and we also performed interaction analysis to reduce the influence of confounding factors and improve the reliability of the conclusions.

Finally, we performed a stratified analysis on the basis of subgroups defined by different covariates to evaluate interactions of the independent associations between fasting blood glucose and urea. We found that in the group with high fasting blood glucose, smoking and alcohol consumption may be two significant modifiers of the relationship between fasting blood glucose and urea. A previous study of healthy Chinese adults revealed that urea levels are lower in smokers than in non-smokers, suggesting that smoking alters urea synthesis or excretion (37). For people who consume alcohol, patients with alcoholism have significantly greater urinary nitrogen and lower nitrogen balance during alcohol consumption (38). However, another study showed that alcohol downregulates urea synthesis in healthy men, which is beneficial for nitrogen preservation (39). The potential mechanisms of these controversies need further study.

This study has the following limitations. First, as a cross-sectional study, causal inferences cannot be made on the basis of the findings of this study. Future longitudinal studies are needed to validate the temporal relationship between fasting blood glucose and urea levels. Second, we only estimated the glomerular filtration rate with the formula rather than the ^99m^Tc-DTPA renogram, which inevitably led to inaccurate results. In addition, while we maximized adjustments using all metabolism-related biomarkers available in the dataset, the absence of intervention data or supplementary biomarkers, such as physical activity levels, protein source and stress is a limitation. Finally, because the participants in the CHNS dataset were mainly from the Chinese population, the results may have certain limitations when applied to other populations. These limitations should be considered in future studies.

## 5 Conclusions

In conclusion, our study suggests an L-shaped association between fasting blood glucose and urea. In non-diabetic individuals, when fasting blood glucose was lower than 4.6 mmol/L, it had a strong negative correlation with urea. The relationship disappeared when the fasting glucose concentration was >4.6 mmol/L.

## Data Availability

Publicly available datasets were analyzed in this study. This data can be found here: http://www.cpc.unc.edu/projects/china.
