# Supplementary material for: L-shaped association between fasting blood glucose and urea in a non-diabetic population
Source: Front Nutr. 2025 Mar 24;12:1504855. doi: 10.3389/fnut.2025.1504855 (PMC11973067; doi:10.3389/fnut.2025.1504855)
Supplement: Supplementary file 1 [file Data_Sheet_1.docx]

Supplementary Material

# Supplementary Figures


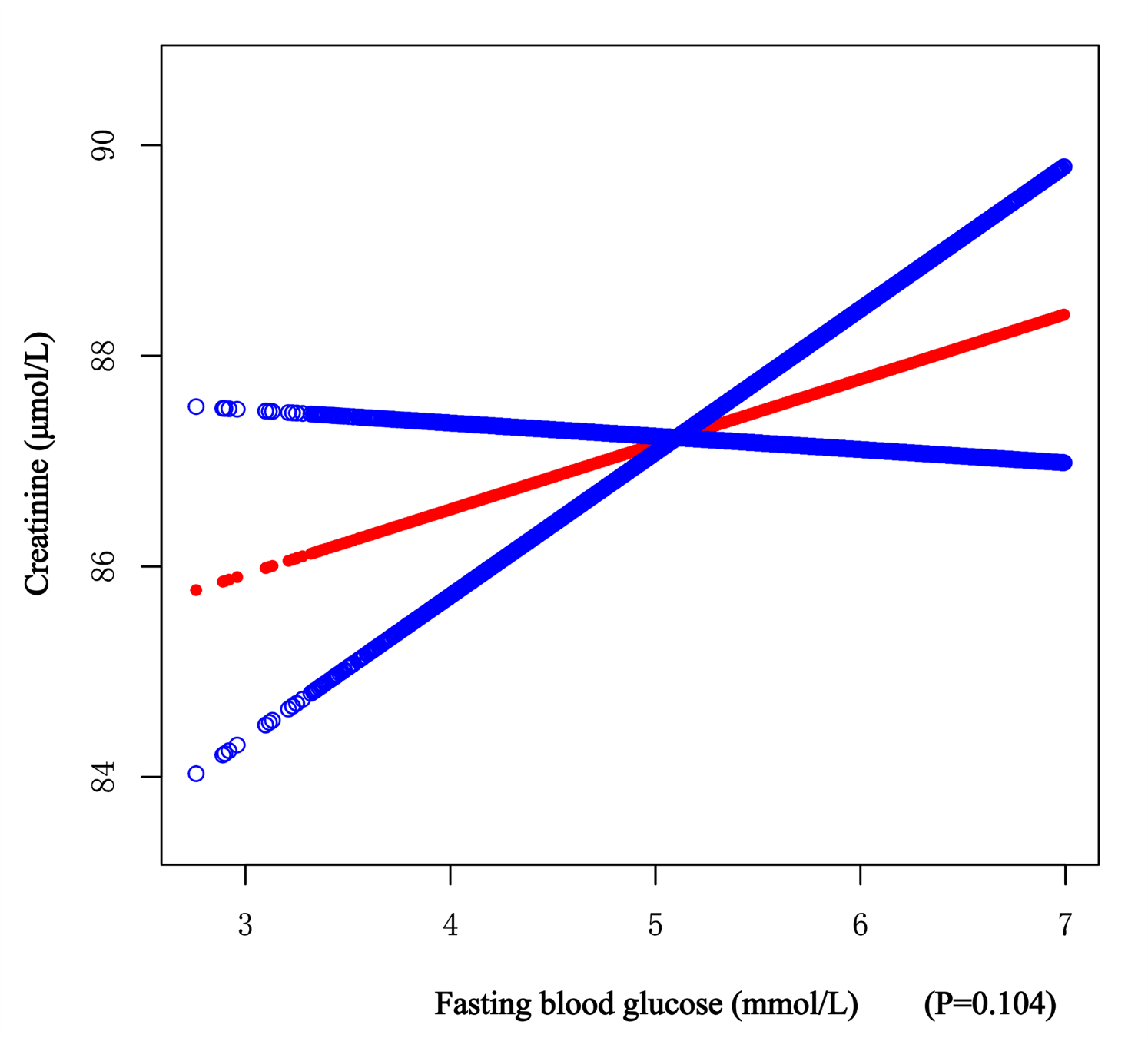


**Supplementary Figure S1.** Smooth curve on association between fasting blood glucose and creatinine. Note: Adjustment factors included age, gender, body mass index, systolic blood pressure, diastolic blood pressure, hypertension, stroke, miocardial infarction, smoking, alcohol consumption, tea consumption, coffee consumption, and soft/sugared fruit drinks consumption, carbohydrate intake, fat intake, dietary protein intake, and calories intake, uric acid, urea, high-density lipoprotein cholesterol, low-density lipoprotein cholesterol, triglyceride, total cholesterol, total protein, and albumin.


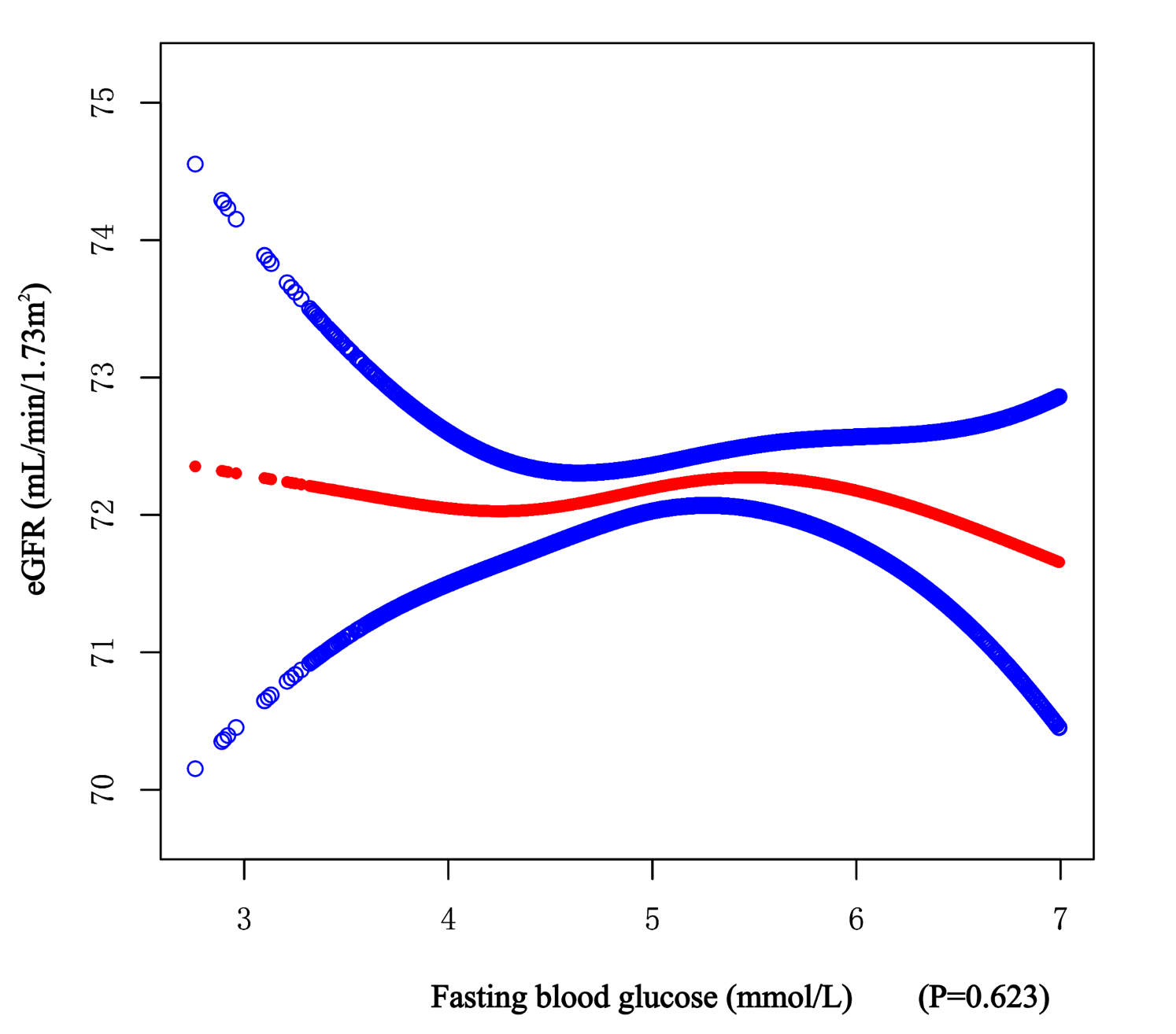


**Supplementary Figure S2.** Smooth curve on association between fasting blood glucose and eGFR. Note: Adjustment factors included age, gender, body mass index, systolic blood pressure, diastolic blood pressure, hypertension, stroke, miocardial infarction, smoking, alcohol consumption, tea consumption, coffee consumption, and soft/sugared fruit drinks consumption, carbohydrate intake, fat intake, dietary protein intake, and calories intake, uric acid, urea, creatinine, high-density lipoprotein cholesterol, low-density lipoprotein cholesterol, triglyceride, total cholesterol, total protein, and albumin; Abbreviations: eGFR = estimated glomerular filtration rate.
